# Distinct inhibitory connectivity motifs could trigger distinct forms of anticipation in the retinal network

**DOI:** 10.1038/s41598-026-49899-w

**Published:** 2026-05-15

**Authors:** Simone Ebert, Bruno Cessac

**Affiliations:** 1 Université Côte d’Azur, Inria Biovision Team and Neuromod Institute, Nice, France; 2 Sorbonne Université, INSERM, CNRS, Institut de la Vision, 17 rue Moreau, Paris, 75012 France; 3 Hertie Institute for AI in Brain Health, University of Tuebingen, Tuebingen, Germany

**Keywords:** Computational biology and bioinformatics, Neuroscience

## Abstract

Motion is an important feature of visual scenes and retinal neuronal circuits selectively signal different motion features. It has been shown that the retina can extrapolate the position of a moving object, thereby compensating sensory transmission delays and enabling signal processing in real-time. Amacrine cells, the inhibitory interneurons of the retina, play essential roles in such computations although their precise function remain unclear. Here, we computationally explore the potential effects of two different inhibitory connectivity motifs on the retina’s response to moving objects, in a simplified model of the retina: feed-forward and recurrent feed-back inhibition. In this model, both motifs can account for motion anticipation with two different mechanisms. Feed-forward inhibition truncates motion responses and shifts peak responses forward via subtractive inhibition, whereas recurrent feed-back coupling evokes excitatory and inhibitory waves with different phases that interfere and shift the response peak. A key difference between the two mechanisms is how the anticipatory peak shift scales with the speed of a moving object. Motion prediction with feed-forward circuits monotonically decreases with increasing speeds, while recurrent feed-back coupling induces tuning curves that exhibit a preferred speed for which motion prediction is maximal.

## Introduction

A visual scene is constantly in motion, not only because our external environment contains moving objects, but also because ourselves and our eyes move. Already in the retina, several cell-types are specialized to detect a variety of motion features such as the global motion of a visual scene versus the local motion of an object^1,2^, looming objects^3–5^ or the direction of motion^6,7^.

In addition to detecting these motion features, the brain has to represent them in real-time. To compensate for delays in neuronal responses, the mouse retina can predict the trajectory of a moving object^8^: When a bar moves across the receptive field of retinal ganglion cells (RGCs), the peak-firing rate of the cell occurs earlier as if the bar is flashed above the receptive field center. This peak shift of the response has been coined “motion anticipation” and implies that, already in the inner retina, cells form a prediction of the future position of a moving object^9^. How such predictive computations emerge from retinal circuitry remains an open question in sensory neuroscience.

This predictive capacity of the retina has been investigated in several experimental and computational studies. First, it has been shown that negative feed-back mechanisms such as gain control^8^ can induce motion anticipation, which can also account for more complex motion extrapolation, such as tracking the position of a moving object in a 2D-plane^10^ or signaling the onset and reversal of moving objects^11,12^. However, the principle of gain control is a rather broad and could rely on many underlying biophysical mechanisms. Other studies provided more mechanistic explanations and showed how amacrine inhibition can contribute to motion anticipation through spatially and temporally displaced feed-forward inhibition^13^, or by forming an anticipatory wave ahead of the stimulus via gap junctions^14–17^, or via lateral recurrent feed-back^18^.

A particularly important dimension of motion anticipation is its dependence on stimulus speed. In order to anticipate a moving object at the correct position, at the correct time, retinal circuits must implicitly encode velocity. A wide range of retinal cell types are tuned to the velocity of a moving object^19–23^, and RGCs can encode object speed for motion processing^24^. In addition, some cells are tuned to a “preferred” speed at which anticipation is maximal, while others maintain a stable anticipation in a wide speed range (0.1–1.0 mm/s, see^8^ or^13^). A recent experimental study has shown that direction selective responses in the retina can be tuned to velocity by distinct inhibitory circuits^25^. Thus, speed-dependent anticipation may as well provide a functional signature of underlying connectivity.

While previous studies demonstrated that amacrine cells can contribute to motion anticipation in several ways, they leave open a key question: How does amacrine cell connectivity affect implementations of motion anticipation? Moreover, recent work suggests that additional predictive mechanisms may act downstream in the visual cortex^26,27^, further emphasizing the need to clarify what computations are already implemented in the retina. Here, we address this question using a deliberately minimal, *linear* model of the retinal inner plexiform layer. Rather than aiming to reproduce the full biological complexity of the retina, our goal is to isolate and compare the computational consequences of two canonical inhibitory connectivity motifs involving amacrine cells: (i) *feed-forward* inhibition and (ii) recurrent *feed-back* inhibition. By restricting the model to three interacting cell types and a small number of parameters, we can directly link network connectivity to dynamical response properties.

We show that these two motifs give rise to distinct forms of motion anticipation, that arise at different processing stages, and differ in their dependence on stimulus speed. Feed-forward inhibition induces anticipatory peak shifts primarily at the level of RGCs and acts most strongly at low speeds. In contrast, recurrent feedback inhibition generates anticipation already at the level of bipolar and amacrine cells and produces a tuning curve with a preferred speed, whose position depends on the strength of recurrent coupling and on the characteristic integration time of the cells.

These results suggest that these distinct inhibitory motifs are not merely alternative implementations of the same computation but instead support qualitatively different predictive regimes. Hence, our findings suggest that diversity in inhibitory connectivity may allow the retina to flexibly set a range of speeds over which motion anticipation is stable and precise.

## Results

### A for motion anticipation via inhibition

**Figure 1:**
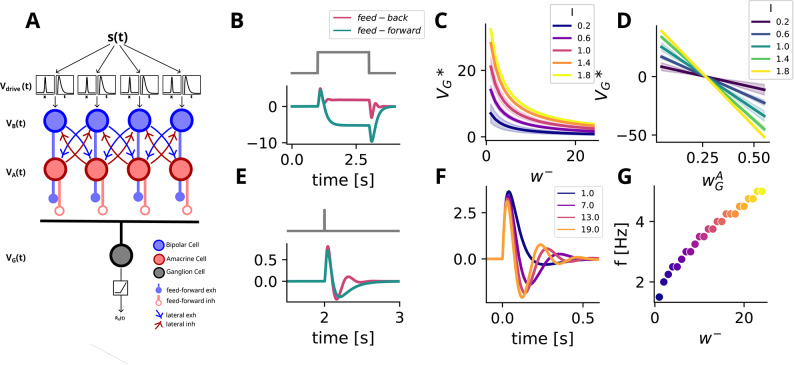
Schematic description of the model and its general response properties. (**A**) The stimulus *s*(*x*, *t*) is fed into a convolution layer that simulates the transformation of the visual input into a neuronal voltage response, $V_{drive}(t)$, for each BC in the network. This convoluted signal is then fed into a network of BCs and ACs, which are reciprocally connected, and pass the synaptic signals $V_B(t)$ and $V_A(t)$ on to neighboring cells of the other type. A third layer of RGCs pools over BCs within their receptive field and integrates inputs $V_B(t)$ into their voltage $V_G(t)$. This voltage is transformed into a firing rate response $R_G(t)$ after rectification. (**B**) Example of step response with both connectivity motifs, feed-back (purple) and feed-forward (green) inhibition. They evoke a similar transient response at the onset of the stimulus, which then decays to a rest state differing between motifs. (**C**) Rest state potential for constant and spatially homogeneous inputs of different amplitudes across recurrent feed-back inhibitory strength $w^-$. (**D**) Rest state potential for constant inputs of different amplitudes across feed-forward inhibitory strength $w^G_A$. (**E**) Example of impulse response with both connectivity motifs. (**F**) Impulse responses for different feed-back inhibitory strengths $w^-$. (**G**) Leading frequency of impulse response varies with feed-back inhibitory strength $w^-$. Same color legend as in (**F**).

We designed a 1-D network model consisting of 3 layers to simulate retinal processing of an incoming stimulus (Fig. 1). Individual bipolar cells (BCs), amacrine cells (ACs) and retinal ganglion cells (RGCs) are simulated as *point neurons* and are characterized by their voltage response, *V*(*t*). The full set of equations describing the model are presented in the Supplementary section .

In the first layer, the spatiotemporal stimulus *s*(*x*, *t*) is convolved with a spatiotemporal kernel, transforming a visual scene into a voltage response $V_{drive}(t)$ (eq. (4)) in BCs. The kernel has a characteristic integration time $\tau _{RF}$, summing up transmission delays in the Outer Plexiform Layer (OPL), and a spatial Gaussian filter representing the receptive field of the BC *i* centered at its position $x_i$. $V_{drive}(t)$ (Eq. (4)) is thus purely evoked by the stimulus. We simulate only the positive center input from photo-receptors. We intentionally omit horizontal cells^28^, as our goal is to isolate the role of inhibitory interactions within the inner retina in shaping motion anticipation. While horizontal cells may also be captured within the feed-forward connections of our model, inhibitory feedback via horizontal cells is diverse and includes non-synaptic mechanisms, which are more complex to simulate and to are out of the scope of this paper.

The second layer of the model simulates BC and AC interactions featured as a set of linear coupled differential equations. The voltage response of each cell type has a characteristic time constant $\tau _B$ (for BCs) and $\tau _A$ (for ACs) (see Eq. (8)). The network consists of two sub-layers of *N* regularly spaced BCs, with index $i = 1...N$, and *N* ACs, $j = 1...N$, which share the same horizontal spatial position $x_{i} = x_j$. The distance between two neighbouring cells is noted $\delta$ (in mm).

The connectivity is simulated through matrices $\Gamma ^{B}_{A}$ and $\Gamma ^{A}_{B}$, which define the connections from BCs to ACs and from ACs to BCs, respectively. Each BC *i* is reciprocally connected to neighboring ACs $j = i-1$ and $j = i+1$ and vice versa. The connections from BCs to ACs are excitatory and have synaptic weight $w^{+} \ge 0$ while connections from ACs to BCs are inhibitory and have synaptic weight $-w^{-} \le 0$. These inhibitory connections back from AC to BC are referred to as feed-back connectivity (Fig. 1A, solid arrows). Although ACs do not receive any direct input from the OPL, they respond to stimuli through BCs input. We do not explicitly model AC receptive fields, but they do have a receptive field resulting from this interaction. Our connectivity scheme is clearly simplified compared to biological lateral connectivity in the retina but affords mathematical analysis. In the discussion we elaborate about these simplifications and how our result can be extrapolated to a more realistic setting.

In the third layer, retinal ganglion cells (RGCs) pool over the voltage responses of bipolar and amacrine cells within a distance $\sigma _G$ corresponding to their receptive field, through weighted linear synapses. The weight $w^{B_i}_{G_k} \ge 0$ describes excitatory connections from BC *i* to RGC *k* and the weight $w^{A_i}_{G_k} \le 0$ describes inhibitory connections from AC *i* to RGC *k*. These connections are referred to as feed-forward connectivity (Fig. 1A, bright lines). We note $W^{{B}_{}}_{{G}_{}}$ (resp. $W^{{A}_{}}_{{G}_{}}$) the connectivity matrix from BCs to RGCs (ACs to RGCs). The dynamics of the RGCs voltage is described by *N* linear differential equations with a shared time constant $\tau _{G}$ (Eq. (10)). Their voltage response is then passed through a non-linear function $N_{G}$ to simulate a firing rate, $R_{G}(t)$ (13).

The dynamics can be summarized and tuned by the time constants $\tau _{RF},\tau _B, \tau _A, \tau _G$, and the synaptic weights $w^+,w^-,w^B_G, w^A_G$ of the model. As shown in^18,29,30^, some of these parameters can be combined to define dimensionless parameters having a deep impact on global dynamics. Especially the product of parameters:

1 $$\begin{aligned} \eta =w^-\,w^+\,\tau _A\,\tau _B, \end{aligned}$$

quantifies the feed-back effect between BCs and ACs.

A frequently observed property of the circuits in the retina is that synaptic connections dynamically adapt during stimulation^31–34^. If synaptic weights are not static but tuned to the visual input, then the parameter $\eta$ also varies with the stimulus and the feed-back strength becomes stimulus dependent.

We explore the effects of inhibitory feed-forward and feed-back connections separately. For simulations with feed-forward connectivity, we set $w^{-} = 0$ to remove feed-back inhibition. For simulations with feed-back connectivity, we set $w^{A}_{G} = 0$. We keep all other parameters the same across the two conditions to isolate the effect of connectivity from the effect of parameter choices.

### General response properties of the different connectivity motifs

In response to a static and spatially homogeneous full-field input, both inhibitory motifs lead to a transient voltage response in RGCs at the onset of the stimulus, which then decays to a rest state (constant voltage) $V_{G}^{*}$ (Fig. 1 B). This rest state depends on the input strength and its analytic form can be easily computed. It strongly differs between the two connectivity motifs. We note $\vec {F}$ the constant stimulus vector, such that a BC with index *i* receives an input $F_i.$

In the network with recurrent feed-back connectivity ($W^{{A}_{}}_{{G}_{}} = 0$), the rest state is:

2 $$\begin{aligned} \vec {V}_{G}^*\,=\, \tau _B \, \tau _{G} \, W^{{B}_{}}_{{G}_{}}.\left( \, \mathscr {I}_N \,+\, \eta \, \Gamma ^{A}_{B} \,\Gamma ^{B}_{A} \, \right) ^{-1}.\vec {F}, \end{aligned}$$

where $\mathscr {I}_N$ is the *N*-dimensional identity matrix. We see that the parameter $\eta$ acts on the rest state which is *divisively* modulated via the inverse matrix $\left( \, \mathscr {I}_N \,+\, \eta \, \Gamma ^{A}_{B} \,\Gamma ^{B}_{A} \, \right) ^{-1}$.

The product $\Gamma ^{A}_{B} \,\Gamma ^{B}_{A}$ corresponds to feed-back from BCs to BCs via ACs and from ACs to ACs via BCs.

For small weights $w^{+}$ and $w^{-}$ the rest state is controlled by $W^{{B}_{}}_{{G}_{}}$, i.e. by the direct input from BCs to RGCs.

As $w^-$ increases, the term $\eta \, \Gamma ^{A}_{B} \,\Gamma ^{B}_{A}$ grows and the feed-back influence gradually dominates, while the rest state decreases in amplitude, following a hyperbola, (Fig. 1 C). Whatever the amplitude of $w^-$, the rest state remains positive. Although the difference between rest states for different input amplitudes decreases with increasing $w^{-}$, recurrent-feed-back coupling maintains a voltage response above 0 which scales with input amplitude even with strong inhibition.

For an RGC with feed-forward inhibition the rest state vector is:

3 $$\begin{aligned} \vec {V}_{G}^*\,=\, \tau _B \, \tau _{G} \, \left( \, W^{{B}_{}}_{{G}_{}} \,+\, \tau _A w^+ W^{{A}_{}}_{{G}_{}}.\Gamma ^{{B}_{}}_{{A}_{}} \, \right) .\vec {F}. \end{aligned}$$

We see that the connections $W^{{A}_{}}_{{G}_{}}$, multiplied by the time constant ($\tau _A$) of inhibition, act now *subtractively* on the rest state, which now linearly scales with input amplitude (Fig. 1 D) where the matrix $\tau _B \, \tau _{G} \, \left( \, W^{{B}_{}}_{{G}_{}} \,+\, \tau _A w^+ W^{{A}_{}}_{{G}_{}}.\Gamma ^{{B}_{}}_{{A}_{}} \, \right)$ acts as scale “factor”. Qualitatively, for small inhibitory weights $W^{{A}_{}}_{{G}_{}}$, the scale factor is big, leading to a strong separation between rest states. As $W^{{A}_{}}_{{G}_{}}$ increases, this separation becomes smaller because the scale factor decreases. When the effect of inhibitory weights increases further, the rest state will eventually become negative.

Given that the RGC voltage is rectified during transformation into firing rate (13), a modulation of the inhibitory strength below this threshold will not yield a change in the RGC spiking output.

In response to a full-field impulse stimulus, the two motifs also qualitatively differ in their response shape (Fig. 1 E). For our set of parameters, feed-forward inhibition leads to a biphasic response profile. The positive and negative phases come from the respective bipolar and amacrine inputs, with a delay corresponding to their respective characteristic times.

In contrast, feed-back inhibition for the same set of parameters can produce a multi-phasic profile into the impulse response. This multiphasic shape comes from damped oscillations in the system, due to the presence of complex frequencies^29,30^. The characteristic frequency of these oscillations depend on the parameter $\eta$^18,29,30^. Figure 1 F,G shows that the leading frequency in these oscillations increases with the strength of recurrent inhibition.

### Connectivity motifs give rise to different mechanisms for motion anticipation

**Figure 2:**
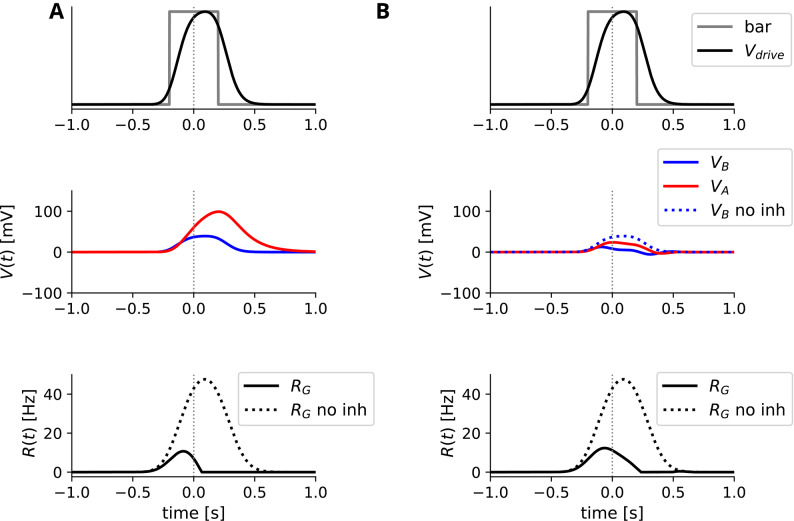
Response at each stage of the model, to a bar moving from left to right at 0.7 *mm*/*s*, for the two connectivity motifs. (**A**) *Feed-forward* connectivity evokes anticipation at the level of RGCs. Upper panel: Bar stimulus (grey) and spatio-temporal convolution yields $V_{drive}$ (Eq. (4)), which simulates the response of the OPL to a visual stimulus. Middle panel: BC (blue) and AC (red) voltage responses to the bar stimulus. Lower panel: RGC firing rate in response to the bar stimulus when inhibition is present (solid black) compared to a purely excitatory feed-forward network (dotted black). (**B**) Recurrent *feed-back* connectivity can evoke anticipation at the level of bipolar cells. Panels show same as in (**A**).

A moving bar with speed *v*, whose center is located at the left edge of the retinal network, $x = 0$ at time $t_0 = 0$, will be at the receptive field center $x_i$ of the downstream bipolar and ganglion cell *i* at $t_{bar_i} = \frac{x_i}{v}$ (see Figure S1 **A**). In our model, the moving bar stimulus first evokes an OPL voltage response called $V_{drive}$ (Eq. (4) and Fig. 2A). The peak in $V_{drive}$ lags behind the stimulus due to the convolution with the temporal kernel (see Figure S1 **A,C**). Cellular integration at each stage of the downstream circuit causes additional delays in the response.

In a purely excitatory network, the response peak of a BC and a RGC, at $t_{B_i}^{peak}$ and $t_{G_i}^{peak}$ respectively, thus always lags behind the center of the bar with increasing delays at each stage of the network (see the dotted lines in Fig. 2). Inhibition can compensate for this delay by truncating the excitatory response, strongly reducing the response amplitude of the network while shifting the response peak forward. If this peak shift becomes larger than the delay introduced by photo-transduction and downstream integration, this corresponds to an *anticipatory response*. In the following sections, we show how the mechanism behind the peak shift differs qualitatively and quantitatively through feed-forward and feed-back connectivity.

#### Feed-forward inhibition implements motion anticipation at the level of Ganglion Cells

With feed-forward connectivity, response delays are first increased during integration into BCs, and are again increased during integration into ACs membrane voltage (Fig. 2A). RGCs then pool over BCs and ACs, while AC inhibition arrives shortly after BC excitation. This delay leads to an initial excitation that is truncated when inhibition starts to rise, and soon fully suppressed. An advancement of the response peak is present (Fig. 2A, last row) compared to a network without inhibition. Thus, feed-forward connectivity anticipates motion via a *substractive* truncation of excitatory inputs. This truncation acts locally: only ACs directly connected to a RGC impact the peak timing.

#### Recurrent feed-back implements motion anticipation already at the level of Bipolar Cells

While the OPL stage remains unchanged, ACs now inhibit BCs shortly after they begin to respond to the visual stimulus. A peak shift is thus induced already in BCs (Fig. 2B).

The propagating bar stimulates BCs, which transmit their response to connected cells via lateral connectivity. The feed-back loops between BCs and ACs create oscillatory responses (as in Fig. 1F), which laterally travel as waves in *both directions* (forward and backward), with a stronger effect forward (in the direction of the stimulus). In this process, each BC can then be viewed as a wave source, triggered by the stimulus, emitting waves that interfere with other waves. The response of a cell is thus a superimposition of its own response to the stimulus and to the lateral waves coming from other BC sources in the network. The influence of other cells exponentially decays with the distance, the closest loops having the strongest impact. Due to the difference in phases, the superimposition of waves creates an offset in the response peak, which can be in advance (anticipation) or delayed. In the setting chosen in this paper, the dominant effect of lateral connectivity is the suppression of the nearest neighbor cell, which truncates the response and is the primary cause of the anticipatory peak shift. The precise timing and amplitude of the response peak depends on the spatio-temporal Fourier spectrum, and also on the speed of the moving bar^29^.

### Tuning to bar speed qualitatively differs in the two connectivity motifs

The network response to a moving bar changes with speed. To illustrate this, we transform the temporal phase shift between response peak and bar position into a spatial measure. We calculate the peak shift for RGCs as $\delta X_{G_i}^{peak} = v \, \delta t_{G_i}^{peak}$ with $\delta t_{G_i}^{peak} = t_{G_i}^{peak} - t_{bar_i}$.

The lag induced by the temporal integration of $V_{drive}$ continuously increases with increasing speed (Figure S1 **A,C**). This is because the bar spends less time in the receptive field with increasing speed (Figure S1 **B**), but the “transduction delay” $\tau _{RF}$ stays constant. The gap between the two times thus increases with increasing speed.

Similarly, the amplitude of the response to the moving bar decreases with increasing speed as the faster bar spends less and less time in the receptive field (Figure S1 **D**).

#### Feed-forward inhibition strongly anticipates slow speeds

**Figure 3:**
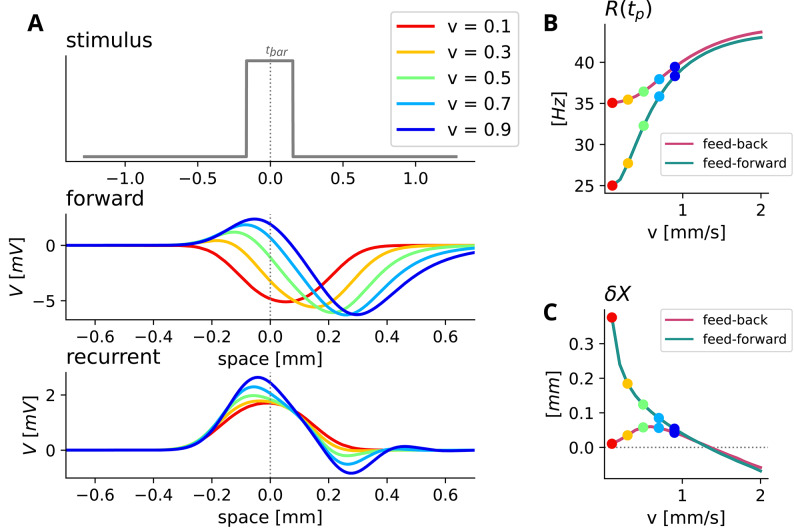
Feed-forward and recurrent feed-back inhibition result in qualitatively different tuning to bar speed. (**A**) Upper: Bar stimulus. Below: Response traces of $V_{G}$ in the feed-forward network (middle) and the recurrent feed-back network (lower). Traces are plotted against the distance of spatial position of the bar center from the RF center at time *t*, motion from left to right. (**B**) Peak amplitude $V(t^{i}_{G})$ plotted against bar speed. (**C**) $\delta X^{i}_{G}$ plotted against bar speed.

Feed-forward inhibition strongly shifts the response peak for the slowest speed tested, so that $\delta X_{G_i}^{peak}$ is large. The peak shift $\delta X_{G}^{peak}$ then decreases with increasing speed. Indeed, truncation always starts when inhibition starts to increase - at a fixed delay to when the bar enters the BC receptive field. The time the bar spends in the receptive field inversely scales with *v* (Figure S1 **B**), thus anticipation inversely scales to bar speed as well and is always maximal for the lowest speed (Fig. 3C). The peak shifts via feed-forward inhibition thus scales with the bar speed along an hyperbola.

Slow bars spend more time in the receptive field and trigger strong responses. This results in a strong suppression of response amplitude for slow speeds, with amplitude increasing as the bar moves faster (Fig. 3B). For very fast speeds, inhibition is too slow to impact the excitatory response while the bar is in a cell’s RF. From this point on, the network fails to anticipate. The amplitudes follow the scaling of $V_{drive}$ and start to decrease (Figure S1 **D**).

#### Recurrent inhibition tunes anticipation to bar speed

In contrast, the response of the recurrent network is *tuned* to the bar speed, such that cells exhibit a preferred speed for which anticipation is *maximal* (Fig. 3A, C).

This tuning arises because moving bars of different speeds induce different wave patterns in the voltage response of RGCs, resulting in differently shaped responses (Fig. 3A, lower panel). Slow speeds trigger slow oscillating responses. As speed increases, responses oscillate faster. Faster oscillations lead to an earlier first peak in the response, the peak shift is bigger. At the same time, the lag via the temporal integration delay increases with increasing speed. Eventually this lag becomes bigger than the increase in anticipatory shift, leading to a tuning curve with a maximally anticipated speed where the advancing shift is strongest relative to the lag.

The peak amplitude tuning to different speeds in the recurrent feed-back case is similar to the feed-forward case, with exceptions for slow speeds (Fig. 3B). Here, the amplitudes remain at a higher level because the amplitudes of excitation and inhibition are recurrently coupled.

### Tuning to bar speed depends on inhibitory strength

The tuning between peak shift and bar speed via recurrent feed-back coupling depends on the parameter $\eta$, the feed-back intensity. A frequently observed property of circuits in the retina is that synaptic connections dynamically adapt during stimulation; connectivity weights are not static during presentation of a visual scene. We thus explored how the tuning to speed in the recurrent network is affected by connectivity weights. For this, we vary $w^-$ and thereby $\eta$.

**Figure 4:**
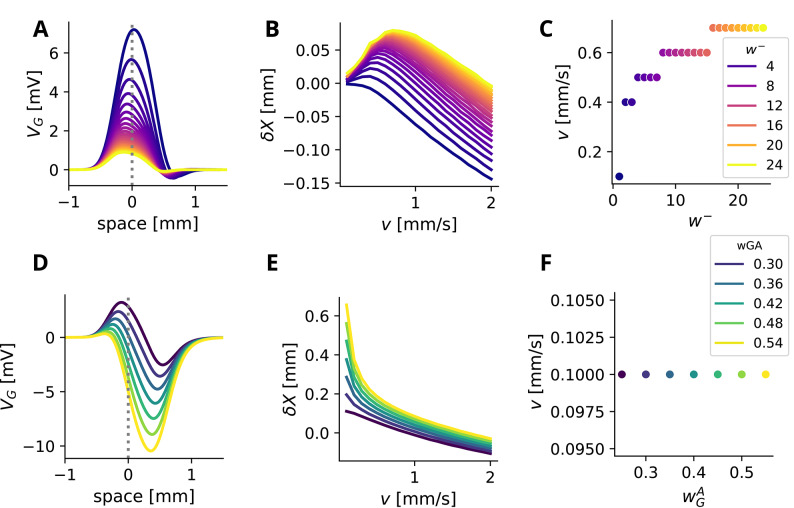
Tuning to bar speed depends on inhibitory strength. Recurrent feed-back (first row) and feed-forward coupling (second row). The color legend applies to the whole row. (**A**) Response for the feed-back coupled network to a moving bar at $v = 0.8 \, mm/s$ for $w^-$ between 0 and 25 Hz. The bar is aligned at the receptive field center at 0. (**B**) Spatial peak shift plotted against bar speed for $w^-$ between 0 and 25 Hz. (**C**) Speed with maximal peak shift plotted against coupling strength. (**D**) Response for the forward-inhibition network to a moving bar at $v = 0.8 \, mm/s$ for $w^{A}_{G}$ between 0 and 0.5 Hz. The bar is aligned at the receptive field center at 0. (**E**) Spatial peak shift plotted against bar speed for $w^{A}_{G}$ between 0 and 0.5 Hz. (**F**) Speed with maximal peak shift during feed-forward inhibition. The fastest speed is always maximally anticipated.

The inhibitory strength of the recurrent feed-back loop $w^-$ impacts the shape of the response: stronger coupling includes stronger and faster oscillations, which result in smaller response amplitude and in a stronger peak shift (Fig. 4A). Over a range of speeds, stronger coupling also leads to a tuning towards faster preferred speeds (Fig. 4B,C).

In the feed-forward connected network, stronger levels of inhibition lead to a stronger suppression of the response and thus to a smaller excitatory response (Fig. 4D). Stronger inhibitory weights lead to stronger overall anticipation with hyperbolic scaling. At low levels of inhibition, the scaling appears linear (Fig. 4E). The speed with maximal anticipation remains always the slowest speed tested (Fig. 4F).

Overall, these results show that the anticipatory mechanism via feed-forward and feed-back responses hold over a wide range of inhibitory weights. In addition, the strength of feed-back inhibition can tune the amount of anticipation to selected speeds, while feed-forward inhibition always anticipates stronger for slower speeds.

### Capturing contrast dependencies via nonlinearities

**Figure 5:**
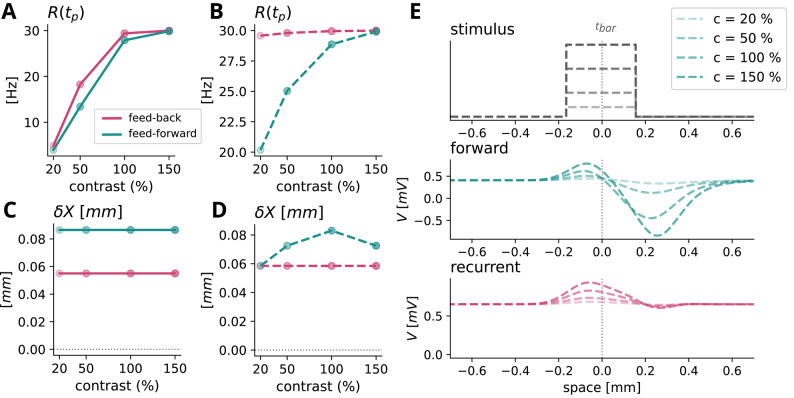
Capturing contrast dependencies via nonlinearities. Recurrent feed-back (purple) and feed-forward coupling (green). The color legend applies to the whole figure. (**A**) Peak amplitude $R(t^{i}_{G})$ plotted against contrast of a moving bar at $v = 0.7 mm/s$ in the linear feed-forward (purple) and feed-back (green) model. A sigmoidal nonlinearity was used to transfrom RGC voltage into firing rate. (**B**) Peak amplitude $R(t^{i}_{G})$ plotted against contrast of a moving bar at $v = 0.7 mm/s$ in the nonlinear feed-forward (purple) and feed-back (green) model. (**C**) $\delta X^{i}_{G}$ plotted against contrast of a moving bar at $v = 0.7 mm/s$ in the nonlinear feed-forward (purple) and feed-back (green) model. (**D**) $\delta X^{i}_{G}$ plotted against contrast of a moving bar at $v = 0.7 mm/s$ in the nonlinear feed-forward (purple) and feed-back (green) model. (**E**) Upper: Bar stimulus at different contrast levels. Below: Response traces of $V_{G}$ in the feed-forward network (middle) and the recurrent feed-back network (lower). Traces are plotted against the distance of spatial position of the bar center from the RF center at time *t*, motion from left to right.

We tested how the two connectivity motifs scale their responses to moving bars across different contrast levels. We simulated the feed-forward and feed-back model responses to moving bars at 0.7 mm/s with stimulus amplitude varying between 20$\%$ and 150$\%$ with respect to the reference contrast in previous simulations ($100\%$). We assumed that the reference contrast used previously lies below saturation, in a range where neuronal responses scale approximately linearly with stimulus amplitude (Fig. 5)

Both connectivity motifs scale the response amplitude to bar contrast similar to experimental observations^8^, where the response amplitude increases with increasing contrast (Fig. 5A). However, both motifs exhibit a constant scaling between anticipation and contrast, conflicting experimental observations where anticipation decreases with decreasing contrast^8^ (Fig. 5C). This is not surprising given that the model we studied so far is linear. Nonlinearities however play an important role for contrast processing in the biological retina. To mimic them, we added sigmoidal nonlinearities to the integration of AC and BC inputs to RGCs, in order to capture thresholding and saturation of synaptic integration (dotted lines in Fig. 5 B,D,E). In the nonlinear case, feed-forward connectivity now induces decreased anticipation with decreasing contrast (Fig. 5 D), as observed experimentally. On the opposite, the nonlinear feed-back model maintains contrast-invariant anticipation. However, different RGC types might exhibit different contrast dependencies in their anticipatory capacity depending on their feature selectivity, and a contrast-invariant anticipatory response may be desirable for upstream computations of object position.

## Discussion

### Response properties to static and dynamic stimuli depend on connectivity patterns

In a constantly moving visual environment, the retina has to reliably signal moving objects at various different speeds. At the same time, it has to anticipate the trajectory of moving objects in order to compensate for transmission delays^8,13,15,16,18^. With a simple and tractable model of the retina, we showed how two different inhibitory connectivity motifs shift the peak response ahead of a moving object, implementing motion anticipation. Feed-back and feed-forward connectivity induce strong differences in the scaling of this anticipatory peak shift with bar speed. Feed-forward inhibition evokes motion anticipation at the level of ganglion cells via subtractive inhibition that vertically propagates through the network. The anticipatory peak shift monotonically decays with speed. Feed-back inhibition evokes motion anticipation also in bipolar and amacrine cells via oscillations in their voltage responses which trigger activity waves laterally traveling through the network. It allows to tune the anticipatory peak shift to object speed. These effects are robust over a relatively wide range of inhibitory synaptic weights. Importantly, increasing the synaptic weight of inhibitory recurrent feed-back shifts this preference to faster speeds. Here, the synaptic weights were modified “by hand”, but synaptic adaptation could do a similar job in the retinal network to dynamically adjust peak position to object speed.

### Comparison to previous models

Previous computational studies of motion anticipation have successfully employed detailed biophysical models^13–15^ or more phenomenological gain-control frameworks^8,10^ and focus on individual retinal ganglion cells. In the present study, we adopted a different perspective where the anticipatory effect of individual cells is the result of a collective response in a dynamical network. To push up this point of view, we use a simplified linear network model that views the retina as a dynamical system^18,29^, lying in between biophysical detail and phenomenological description. It allowed us to isolate how network topology shapes response properties such as the speed dependence of motion anticipation and to study network interactions shaping individual cellular responses.

### Response properties to spatio-temporal stimuli can inform us about the upstream circuitry

We showed how response properties of RGCs to static and moving stimuli can differ due to the connectivity of the upstream network. Examining response properties of different RGC types to these stimuli experimentally could thus hint us to how their upstream circuits are wired.

Anticipation scaling with speed has been shown behave qualitatively different in different RGC types^8^. Some scale more hyperbolic, as would result from feed-forward inhibition in our model, while others show a maximally anticipated speed, as in our feed-back simulations. Yet, other cells exhibit constant anticipation across a range of speed, which could indicate more elaborated mechanisms (see below).

It thus seems likely that different cell-types could employ different mechanisms for motion processing. A combination of feed-forward and feed-back inhibition could also give rise to more elaborate tuning curves.

ACs play central roles in many retinal computations^34–39^, but the precise role of their diversity and complex circuitry are only starting to be understood^40,41^. With a detailed understanding of how different connectivity motifs shape retinal responses, analyzing these properties in retinal cell-types might help to inform us about their upstream circuitry. Especially, oscillatory responses and spatiotemporal frequency preferences could generalize receptive field characterizations.

### Speed representations via dynamic feed-back circuits

Recent studies suggest that dynamic adaptation of retinal circuits play important roles in shaping retinal computations^42–45^, and that dynamic inhibitory synapses adapting to the stimulus frequency can implement temporal pattern recognition in the retina^46^.

The retinal network could also dynamically tune its connectivity weights to the speed of a moving bar via short-term plasticity. This could implement a stable anticipatory signal across a range of speeds, as observed in^8^. One could imagine that inhibitory synapses in the network depress in response to the strong activation by slow speed, which in turn would shift maximal anticipation towards slow speed, thereby stabilizing anticipation across a range of speeds. In addition, especially the recurrent-feed-back circuit is not only characterized by the connectivity weight but by the product $w^- w^+ \tau _A \tau _B$. Therefore, modifying the characteristic times can also impact the response to moving objects and could also occur via dynamically adapting synapses.

### Limitations

The model presented here simplifies the biology of the retinal networks at many stages to allow dissection and mathematical tractability.

The biggest simplification is that the model is linear. While this setup certainly lacks realism compared to the biological retina, it shows that nonlinearities are not necessarily needed to account for complex response properties such as motion anticipation. We showed the main results remain qualitatively similar if the synaptic connection from BC and ACs onto RGCs are rectified, see Supplementary Figure S3. The effect of nonlinearities at feed-back stages in the model on the response to moving stimuli remains to be studied in detail, as low voltage thresholding would limit the effect of ACs on BCs, thereby limiting the peak shift in anticipation.

More generally, one can ask about the genericity of the model and its robustness in terms of connectivity and parameter variations. The model structure is natural and corresponds to what was recently called “the standard model of the retina”^47^, with two main differences: our model is linear and it considers feed-back connectivity. To systematically study the robustness to changes in the model (more complex connectivity, parameter variations), a mathematical study of voltage dynamics and their dependence on such changes can be provided. Especially, the effect of connectivity can be more systematically addressed via spectral studies (eigenvalues and eigenvectors of the flow) precisely because the model is linear. In addition, feed-forward and recurrent feedback inhibition are not mutually exclusive and may act simultaneously within the same circuit. In our model, the combination of these effects corresponds to tuning simultaneously and independently the parameter $w^-$ (feedback), and $w^A_G$ (feedforward). A systematic study is beyond the scope of this paper, and more generally beyond a purely numerical study. The parameter space also includes $\tau _A, \tau _B, w^+$ and others and is too big to explore numerically. This is the object of a forthcoming mathematical paper.

In this study, we simulated all cells as point neurons, which are divided into bipolar, amacrine and ganglion cells by the network architecture and time constants but otherwise share response properties. We cannot realistically reference the circuits analyzed here to specific and retinal cell types but rather focus on general effects of feed-back versus feed-forward connectivity. Our bipolar cells have an RF size of $50 \mu m$ based on^11^, which seems reasonable for ON BCs^48^. Amacrine cell RF sizes are not explicitly modeled but are inherited form the BCs which innervate them. Given nearest neighbors connectivity in our model, the ACs modeled here could be narrow-field ACs, but also wide-field cells with more localized processing units. It is known that A17 amacrine cells form reciprocal synapses with rod-bipolar cells that affect local temporal response properties^49^, but how much the signal spreads laterally in this circuit is not clear.

We refer to inhibitory interneurons as amacrine cells and omit horizontal cells. Indeed, horizontal cells use more complicated and diverse often non-synaptic mechanisms to feedback onto photo-receptors^50^ and are less likely to induce oscillations leading to motion anticipation in our model.

Nevertheless, the inhibitory units in our model could partially capture functional aspects of horizontal cell in the context of feed-forward inhibition.

Finally, future analyses could target a more diverse set of moving stimuli such as coherent or differential moving gratings, and also dissect the consequences of the two connectivity motifs on noise tolerance.

## Methods

### Parameter calibration

Model parameters can be divided into two categories: (i) fixed, structural parameters constrained by previous studies and (ii) dynamical parameters and scaling factors tuned to reproduce qualitative response properties. Structural parameters such as receptive field sizes, cell spacing, number of cells, and ganglion cell pooling width were fixed to values used in previous spatiotemporal convolution models of retinal motion processing^18,29^, themselves grounded in experimental studies^8,11^. The cell spacing of $5 \mu m$ is based on^11^, and seems realistic for several BC types^51^. The number of cells N was chosen to be large enough to limit boundary effects.

We adjusted the dynamical parameter (time constants and connectivity weights) within physiologically plausible ranges to reproduce qualitative features of experimentally recorded responses (Figure S2). Experimental data were obtained from multi-electrode array recordings in dissected retinas of adult mice, using moving bar stimuli with width $b = 160~\mu$m and speed $v = 0.7~\mathrm {mm/s}$. Spatial and temporal receptive fields were estimated via spike-triggered average (STA) analysis using white-noise stimulation. For details experimental protocol, see^52^.

Calibration of dynamical parameters and scale factors were targeted to match one experimentally recorded cell which exhibited motion anticipation. Specifically, time constants and connectivity weights were set such that the impulse response of the model fits to the temporal profile of the STA. Scale factors were chosen such that the firing rate in response to a moving bar was matched. Parameter adjustment was performed manually to match these qualitative response features rather than to achieve a precise quantitative fit.

All parameter values are listed in Table 1.

### Parameter values

**Table 1:** Parameter values used in simulations, if not stated otherwise in the text.

Parameter	Value	Unit	type
$\sigma _{B}$	0.05	mm	Structural^11^
$\sigma _{G}$	0.065	mm	Structural^11^
$\delta$	0.005	mm	Structural^11^
$\tau _{RF}$	0.04	s	Structural^11^
$\tau _{B}$	0.08	s	Dynamic
$\tau _{A}$	0.15	s	Dynamic
$\tau _{G}$	0.01	s	Dynamic
$w^{-}$	-10.0	Hz	Dynamic
$w^{+}$	10.0	Hz	Dynamic
$w^B_G$	0.8	Hz	Dynamic
$w^A_G$	-0.4	Hz	Dynamic
$s_{G}$	5	Hz mV$^{-1}$	Scaling
$\theta _{G}$	0.0	mV	Scaling
$a_{mV}$	20.0	nS$^{-1}$	Scaling
*dt*	0.001	s	–
stimulus intensity	1.0/0.1	pA	Scaling
N	512	–	Structural

## Model implementation

The model was implemented using the Brian2 Python toolbox^53^. All model equations are given in the Supplementary.

## Supplementary Information

Supplementary Information 1.

Supplementary Information 2.

Supplementary Information 3.

## Data Availability

No external datasets were used in this study. All data used in this study are generated through simulations. The code required to reproduce these simulations, as well as all analysis and figure-generation scripts, is publicly available at https://doi.org/10.5281/zenodo.19454849and https://github.com/simoneeb/motion_anticipation_network#.
