# Supplementary material for: Distinct inhibitory connectivity motifs could trigger distinct forms of anticipation in the retinal network
Source: Sci Rep. 2026 May 15;16:22427. doi: 10.1038/s41598-026-49899-w (PMC13376537; doi:10.1038/s41598-026-49899-w)
Supplement: Supplementary file 2 — Supplementary Information 2. [file 41598_2026_49899_MOESM2_ESM.pdf]

Supplementary Material

Model Equations

We consider a 1-D moving bar stimulus $s(t)$ (in pA) with a speed v (in mm/s) and width $2b$ (in mm). We simulate the voltage input from the OPL to a bipolar cell i with a receptive field center located at x_i via a spatiotemporal convolution:

$$V_i^{drive}(t) = a_{mV} \int_{-\infty}^{t} \int_{0}^{D} \mathscr{K}(x_i - x, t - u) s(x,u) dx du \tag{4}$$

where the kernel is separated:

$$\mathscr{K}(x,t) = \mathscr{K}_T(t)\, \mathscr{K}_S(x). \tag{5}$$

The temporal profile is given by:

$$\mathscr{K}_T(t) = \frac{t}{\tau_{RF}^2} e^{-\frac{t}{\tau_{RF}}} \tag{6}$$

where τ_{RF} is the characteristic integration time of the OPL. The spatial kernel is a Gaussian:

$$\mathscr{K}_S(x) = \frac{e^{-\frac{(x-x_i)^2}{2\sigma_B^2}}}{\sqrt{2\pi}\sigma_B} \tag{7}$$

where σ_B parametrizes the size of the receptive field center (in mm) of cell i with position x_i and a_{mV} is a scale factor with unit nS^{-1}.

We then consider a retinal network spanning a 1-D plane with N = 512 BCs positioned at x_i and N = 512 ACs with the same spatial location. Cells cover a distance $D = 2.56$ mm and are spaced by $\delta = 0.005$ mm. Each cell is characterized by its membrane potential V_{B_i} and V_{A_j} respectively. The dynamics of the cells is ruled by the dynamical system:

$$\begin{cases} \frac{dV_{B_i}}{dt} = -\frac{V_{B_i}}{\tau_B} - w^- \sum_{j=1}^{N} \Gamma_{B_i}^{A_j} V_{A_j} + F_i(t), \quad i = 1 \ldots N \\ \frac{dV_{A_j}}{dt} = -\frac{V_{A_j}}{\tau_A} + w^+ \sum_{i=1}^{N} \Gamma_{A_j}^{B_i} V_{B_i}, \quad j = 1 \ldots N. \end{cases} \tag{8}$$

The connectivity is simulated via the matrices Γ_A^B and Γ_B^A, which define the connections from BCs to ACs and from ACs to BCs, respectively. Each BC i projects onto ACs $j = i-1$ and $j = i+1$ and vice versa such that $\Gamma_{A_{i-1}}^{B_i} = \Gamma_{A_{i+1}}^{B_i} = 1$. All other entries are set to 0. We assume null boundary conditions. Connections from BCs to ACs are excitatory and have a synaptic weight $w^+ \geq 0$ while connections from ACs to BCs are inhibitory and have a synaptic weight $-w^- \leq 0$. The connectivity between BCs and ACs is symmetric, $\Gamma_A^B = \Gamma_B^A$. This is mathematical choice commented in[53] allowing to avoid linear instabilities in the system, as the eigenvalues of the linear system always have a negative real part with this condition.

The term:

$$F_i(t) = \frac{V_i^{drive}}{\tau_B} + \frac{dV_i^{drive}}{dt}, \tag{9}$$

is the stimulus driven input into BCs. It takes this form to ensure that $V_{Bi} = V_i^{drive}$ in the absence of ACs coupling

Finally, a layer of N = 512 RGCs is added, obeying the differential equations:

$$\frac{dV_{G_k}}{dt} = -\frac{V_{Gk}}{\tau_G} + \sum_{i=1}^{N} W_{G_k}^{B_i} V_{B_i}(t) + \sum_{j=1}^{N} W_{G_k}^{A_j} V_{A_j}(t), \quad k = 1 \ldots N, \tag{10}$$

where each RGC k pools over the BCs layer with Gaussian weights $W_{G_k}^{B_i}$, centered at the RGC's position x_k (same as BC and AC position), and a width σ_G in mm. The scale factor $w_G^B > 0$ (in Hz) determines the overall strength of synapses from BCs to RGCs.

$$W_{G_k}^{B_i} = w_G^B e^{-\frac{(x_i - x_k)^2}{2\sigma_G^2}}. \tag{11}$$

In the feed-forward network, each RGC pools as well over ACs with the same distribution. The synaptic strength is scaled by $w_G^A < 0$ (in Hz):

$$W_{G_k}^{A_j} = w_G^A e^{-\frac{(x_j - x_k)^2}{2\sigma_G^2}}. \tag{12}$$

In the last step, the RGC voltage V_{G_k} is transformed into a firing rate:

$$R_{Gk} = \mathcal{N}(V_{Gk}, \theta_G), \tag{13}$$

via the piecewise-linear function:

$$\mathcal{N}(V) = \begin{cases} s_G(V - \theta), & \text{if } V \geq \theta \text{ ;} \\ 0, & \text{otherwise.} \end{cases} \tag{14}$$

For simulations with feed-forward connectivity, we set $w^- = 0$ to remove feed-back inhibition. For simulations for feed-back connectivity, we set $w_G^A = 0$.

Supplementary Figures

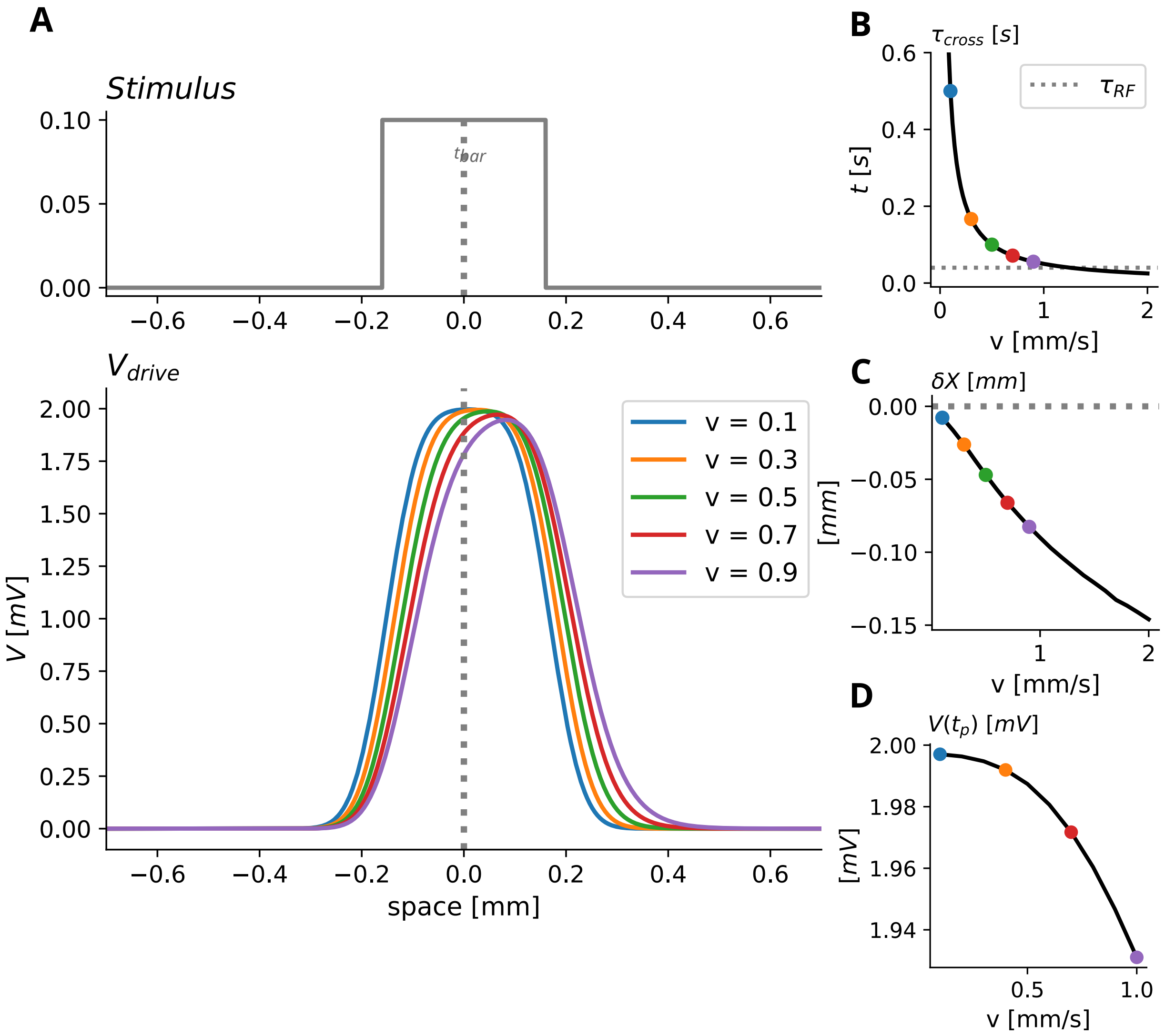

Figure S1. Photo-transduction layer introduces a lag between response peak and bar middle which increases with the speed of the bar A. Upper: Bar stimulus. **Lower**: Response traces of V_{drive} to bars moving at speeds between 0.1 and 1.0 mm/s. Traces are plotted against the distance of spatial position of the bar center from the RF center at time t, motion from left to right. **B.** τ_{cross} plotted against bar speed. τ_{RF} is indicated by the grey dotted line. **C.** δX^i_{drive} plotted against bar speed. **D.** Peak amplitude $V(t^i_{drive})$ plotted against the bar speed.

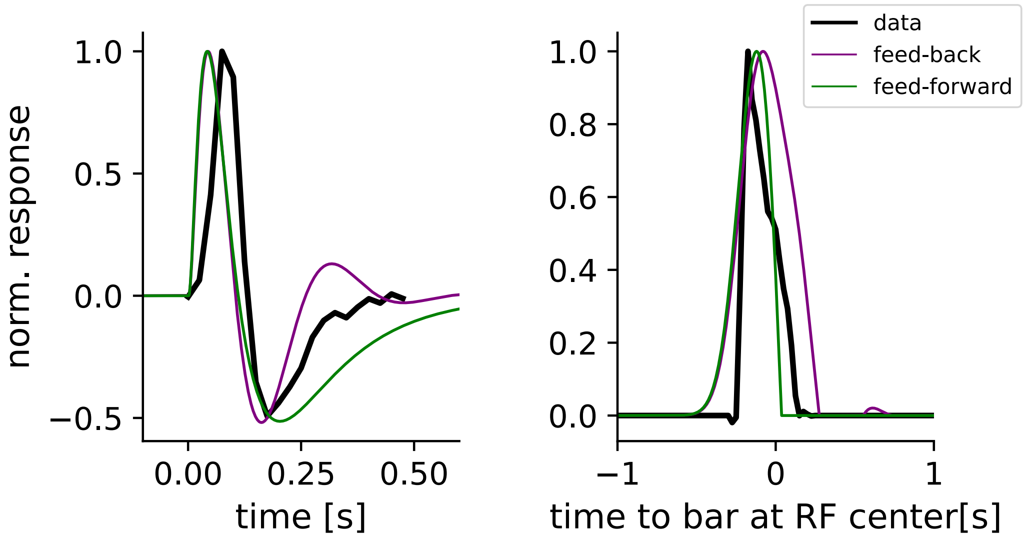

Figure S2. **Model responses optimized to experimental data from of a recorded RGC which anticipates motion. A. Experimental temporal STA** of the RGC and impulse response fit of the model (Courtesy of Olivier Marre and Thomas Buffet). **B. Firing rate** in response to moving bar at 0.7 mm/s and simulations of the network, aligned at t=0 to when the bar is at the center of the receptive field (dotted line). Amplitudes are normalized for comparison.

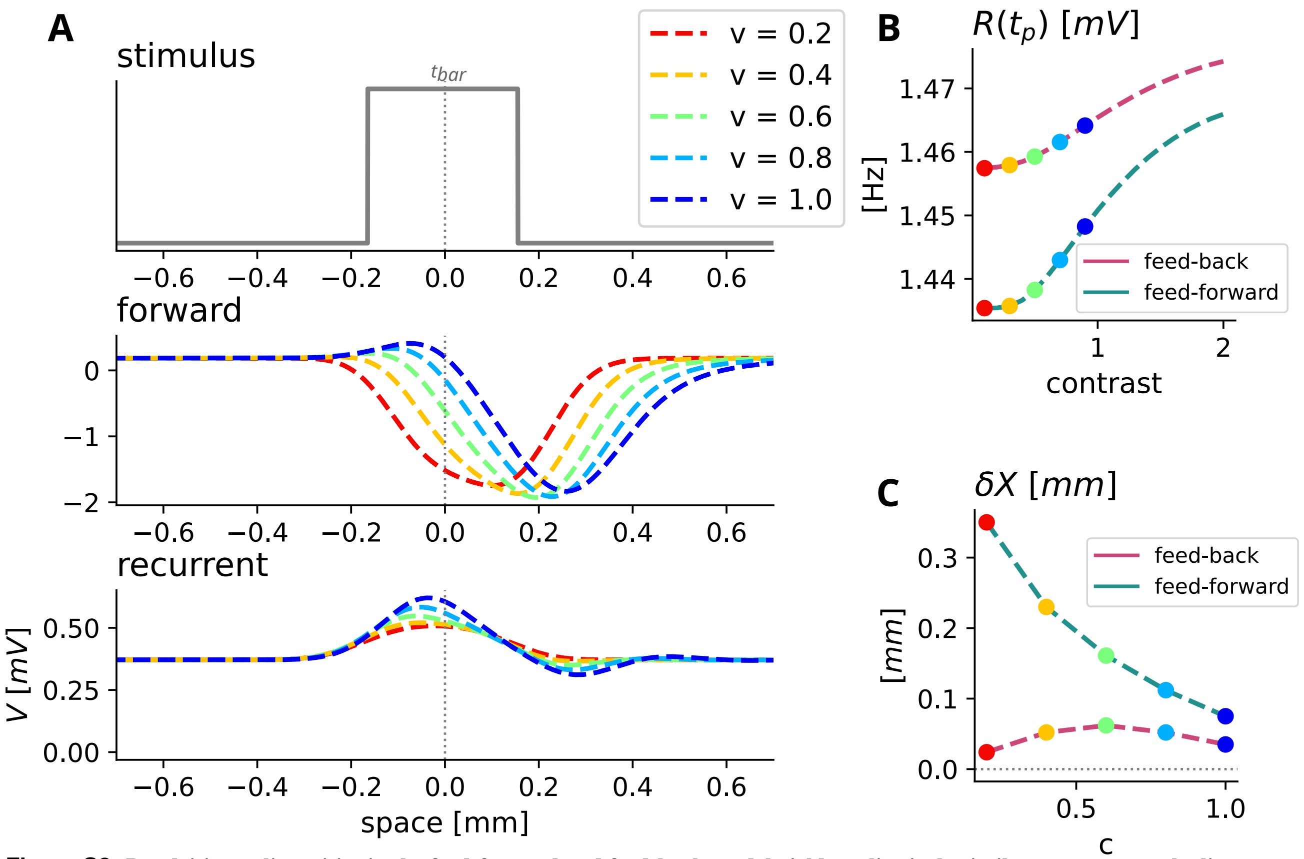

Figure S3. **Dendritic nonlinearities in the feed-forward and feed-back model yield qualitatively similar responses as the linear models. A. Upper**: Bar stimulus. **Below**: Response traces of V_G in the feed-forward network (middle) and the recurrent feed-back network (lower). Traces are plotted against the distance of spatial position of the bar center from the RF center at time t, motion from left to right.
